# The prognostic value of JUNB-positive CTCs in metastatic breast cancer: from bioinformatics to phenotypic characterization

**DOI:** 10.1186/s13058-019-1166-4

**Published:** 2019-08-01

**Authors:** Galatea Kallergi, Vasileia Tsintari, Stelios Sfakianakis, Ekaterini Bei, Eleni Lagoudaki, Anastasios Koutsopoulos, Nefeli Zacharopoulou, Saad Alkahtani, Saud Alarifi, Christos Stournaras, Michalis Zervakis, Vassilis Georgoulias

**Affiliations:** 1 Laboratory of Τumor Cell Βiology, Medical School, University of Crete, Heraklion, Greece; 2 Department of Biochemistry, Medical School, University of Crete, Voutes, 70013 Heraklion, Crete Greece; 3 Department of Oncology, Hematology, Rheumatology, Immunology and Pulmology, University Hospital, Tübingen, Germany; 4 Computational BioMedicine Laboratory, Institute of Computer Science, Foundation for Research and Technology, Heraklion, Greece; 5 Digital Image and Signal Processing Laboratory, School of Electrical and Computer Engineering, Technical University of Crete, Chania, Greece; 6 Department of Pathology, University General Hospital of Heraklion, Heraklion, Crete Greece; 7 Department of Zoology, Science College, King Saud University, Riyadh, Saudi Arabia; 8 Hellenic Oncology Research Group (HORG), Athens, Greece

**Keywords:** Breast cancer, CTCs, JUNB, CXCR4, Bioinformatics

## Abstract

**Background:**

Circulating tumor cells (CTCs) are important for metastatic dissemination of cancer. They can provide useful information, regarding biological features and tumor heterogeneity; however, their detection and characterization are difficult due to their limited number in the bloodstream and their mesenchymal characteristics. Therefore, new biomarkers are needed to address these questions.

**Methods:**

Bioinformatics functional enrichment analysis revealed a subgroup of 24 genes, potentially overexpressed in CTCs. Among these genes, the chemokine receptor CXCR4 plays a central role. After prioritization according to the CXCR4 corresponding pathways, five molecules (JUNB, YWHAB, TYROBP, NFYA, and PRDX1) were selected for further analysis in biological samples. The SKBR3, MDA-MB231, and MCF7 cell lines, as well as PBMCs from normal (*n* = 10) blood donors, were used as controls to define the expression pattern of all the examined molecules. Consequently, 100 previously untreated metastatic breast cancer (mBC) patients (*n* = 100) were analyzed using the following combinations of antibodies: CK (cytokeratin)/CXCR4/JUNB, CK/NFYA/ΥWHΑΒ (14-3-3), and CK/TYROBP/PRDX1. A threshold value for every molecule was considered the mean expression in normal PBMCs.

**Results:**

Quantification of CXCR4 revealed overexpression of the receptor in SKBR3 and in CTCs, following the subsequent scale (SKBR3>CTCs>Hela>MCF7>MDA-MB231). JUNB was also overexpressed in CTCs (SKBR3>CTCs>MCF7>MDA-MB231>Hela). According to the defined threshold for each molecule, CXCR4-positive CTCs were identified in 90% of the patients with detectable tumor cells in their blood. In addition, 65%, 75%, 14.3%, and 12.5% of the patients harbored JUNB-, TYROBP-, NFYA-, and PRDX-positive CTCs, respectively. Conversely, none of the patients revealed YWHAB-positive CTCs. Interestingly, JUNB expression in CTCs was phenotypically and statistically enhanced compared to patients’ blood cells (*p* = 0.002) providing a possible new biomarker for CTCs. Furthermore, the detection of JUNB-positive CTCs in patients was associated with poorer PFS (*p* = 0.015) and OS (*p* = 0.002). Moreover, JUNB staining of 11 primary and 4 metastatic tumors from the same cohort of patients revealed a dramatic increase of JUNB expression in metastasis.

**Conclusions:**

CXCR4, JUNB, and TYROBP were overexpressed in CTCs, but only the expression of JUNB was associated with poor prognosis, providing a new biomarker and a potential therapeutic target for the elimination of CTCs.

**Electronic supplementary material:**

The online version of this article (10.1186/s13058-019-1166-4) contains supplementary material, which is available to authorized users.

## Background

The malignant nature of circulating tumor cells (CTCs) has been recently demonstrated using xenograft models [1, 2], and it is widely accepted that these cells are responsible for tumor metastasis. CTCs have been detected in several tumor types, including breast, prostate, colorectal, non-small cell lung cancer (NSCLC), and small cell lung cancer (SCLC) [3–8]. Their detection has been associated with poor patients’ clinical outcome, irrespectively of the disease stage. In addition, the presence of CTCs before and after the completion of adjuvant chemotherapy in breast cancer patients is associated with poor clinical outcome [6, 9, 10].

Several studies have shown that CTCs present phenotypic and molecular differences from the corresponding primary tumor cells [11–13] and their changes in response to treatment could provide a useful alternate to solid biopsy [13, 14]. In addition, their detection and enumeration can give useful information regarding patient’s prognosis providing a surrogate marker for treatment efficacy [15–17]. Nevertheless, it is well known that chemotherapy cannot effectively eliminate all CTCs; therefore, the identification of new molecules on these cells could offer new therapeutic targets.

Furthermore, only a subgroup of patients with detectable CTCs will eventually relapse. This is attributed to the fact that some of the identified CTCs are destined to die [18]. Therefore, only a proportion of the detected tumor cells have metastatic potential; however, it is currently unknown which is the dangerous phenotype.

CTCs hold epithelial and/or mesenchymal (EMT) characteristics as well as stem cell features [19–21], and the acquisition of an EMT phenotype makes them invisible with common detecting platforms, based on the expression of epithelial markers. Consequently, there is an urgent need for new biomarkers to improve the identification and characterization of these cells.

Bioinformatics analysis is a new tool, based on Gene Expression databases, which can provide information about a number of genes-proteins that could potentially be upregulated in CTCs. Thus, the aim of the current study was the identification of novel biomarker/therapeutic targets in CTCs isolated from metastatic breast cancer patients based on bioinformatics analysis.

According to this analysis, CXCR4 is the most important molecule, potentially overexpressed in CTCs, while JUNB, YWHAB, TYROBP, NFYA, and PRDX1 were also found to be related to CXCR4 in different pathways and they could be potentially upregulated in CTCs [22].

**Table 1:** Patient characteristics

Metastatic breast cancer	
No. of patients enrolled—100	
Age, years	Hormone receptor status
Median, range 59 (66–81)	ER-positive/PR-positive—45 (45%)
ECOG performance status	ER-positive/PR-negative—16 (16%)
	ER-negative/PR-positive—5 (5%)
0—66 (66%)	ER-negative/PR-negative—18 (18%)
1—23 (23%)	Unknown—16 (16%)
2—6 (6%)	No. of disease sites
Unknown—5 (5%)	1—51 (51%)
Histology	2—21 (21%)
Ductal—75(75%)	3—11 (11%)
Lobular—10 (10%)	≥ 4—2 (2%)
Other—9(9%)	Unknown—15 (15%)
Unknown—6 (6%)	Visceral disease
Menopausal status	Yes—37 (37%)
Premenopausal—26 (26%)	No—50 (50%)
Perimenopausal—12 (12%)	Unknown—13 (13%)
Postmenopausal—53 (53%)	Triple negative (ER negPRnegHER2neg)
Unknown—9 (9%)	11 (11%)
HER2 status	
HER2 negative—64 (64%)	
Her2 positive—18 (18%)	
Unknown—18 (18%)	

It is important that CXCR4 has been found to be a prognostic marker in various tumor types, including BC [23]. It is upregulated in tumor tissue as compared to normal tissues. It has also been reported that CXCR4 is important for CTC-seeding and metastatic potential in early-stage BC of node-positive patients. Its expression was related to epithelial-mesenchymal transition (EMT), hence, associated with an acutely malignant phenotype [24].

JUNB has been also associated to invasion/metastasis in solid tumors including BC [25–27] and represented as an important target in diseases associated with EMT, including cancer and fibrosis [28, 29]. It has been implicated in the earliest events of the development of resistance to kinase inhibitors in BC [30]; however, the expression and the prognostic values of this transcription factor in CTCs have not been studied. Furthermore, the rest of the examined molecules (YWHAB, TYROBP, NFYA, and PRDX1) have been related to cancer progression and invasion [31–35], but to the best of our knowledge, they have not been studied in CTCs.

## Methods

### Dataset integration and bioinformatics analysis

We collated an ensemble of nine human genome microarrays (GSE22820; GSE19783; GSE31364; GSE9574; GSE18672; GSE27562; GSE16443; GSE15852; GSE12763), from the Gene Expression Omnibus (GEO) database [36]. The integration of the datasets followed the process described in the literature [22], resulting in 498 primary breast cancer tissues and 124 blood samples from breast cancer patients, as well as 104 normal breast tissue samples and 85 peripheral blood samples from healthy donors. Three comparative analyses were performed in the pooled set of samples, using the “Significance Analysis of Microarrays” (SAM) [37] as the feature selection approach of choice with the siggenes package of R/Bioconductor using the same parameters for all genes entries, in order to reveal comparison-specific differentially expressed genes. The false discovery rate (FDR) [38] was used as the criterion for determining the set of genes that exhibit differential expression with critical value set to 0.01 for all comparisons.

### Patient samples and cytospin preparation

One hundred newly diagnosed and treatment-naïve patients with metastatic breast cancer (mBC) were enrolled in the study. All patients were enrolled in one center (Department of Medical Oncology, University General Hospital of Heraklion, Crete, Greece) and were treated with front-line chemotherapy, according to the national guidelines regardless of the CTCs’ results. Patients’ characteristics are shown in Table 1. In addition, 10 female normal blood donors were also included as controls. All blood samples were obtained at the middle of vein puncture, after the first 5 ml of blood was discarded. These precautions were undertaken in order to avoid contamination of the blood sample with epithelial cells from the skin during sample collection. All patients gave their informed consent to participate in the study, which has been approved by the Ethics and Scientific Committees of our Institution.

**Table 2:** Mean intensity of JUNB, CXCR4, TYROBP, PRDX1, NFYA, and YWHAB in breast cancer cell lines, PBMCs, and CTCs

Mean intensity	Normal PBMCs	Patients PBMCs	SKBR3	MCF7	MDA-MB 231	Hela	Int CTCs
JUNB	5.2 ± 0.15	4.4 ± 0.11	26.1 ± 1.8	8.9 ± 0.56	6.6 ± 0.15	6.2 ± 0.65	9.6 ± 6.3
CXCR4	10.62 ± 1.01	25.4 ± 1.59	168.8 ± 8.25	13.86 ± 0.51	10.22 ± 0.7	26.85 ± 2.69	33.57 ± 9.25
TYROBP	5.17 ± 0.67	4.82 ± 0.21	3.97 ± 0.13	3.61 ± 0.1	5.08 ± 0.09	3.49 ± 0.24	8.89 ± 3.53
PRDX1	15.9 ± 3.62	34.73 ± 9.69	3.53 ± 0.22	6.25 ± 0.18	22.01 ± 1.64	4.83 ± 0.00	29.34 ± 24.65
NFYA	5.91 ± 0.52	6.08 ± 0.25	3.95 ± 0.23	4.93 ± 0.09	7.26 ± 0.15	5.41 ± 0.18	5.20 ± 1.36
YWHAB	26.13 ± 10.10	0 ± 0	4.04 ± 0.15	7.60 ± 0.12	26.45 ± 3.52	8.20 ± 0.4	5.70 ± 0.28

Twenty milliliters of blood in EDTA was obtained from all patients, and peripheral blood mononuclear cells (PBMCs) were isolated with Ficoll-Hypaque density gradient (*d* = 1077 g/mol) centrifugation at 1800 rpm for 30 min. PBMCs were washed three times with PBS and centrifuged at 1500 rpm for 10 min. Aliquots of 500,000 cells were cyto-centrifuged at 2000 rpm for 2 min on glass slides. Cytospins were dried up and stored at – 80 °C. Two slides with 10^6^ cells in total were analyzed from each patient for every molecule and for CK/CD45 staining. This number of PBMCs according to previous studies of our group could be representative of the characterization of CTCs in patients’ blood [18, 19, 39].

### Cell cultures

All cell lines were obtained from ATCC (American Type Culture Collection, USA) and used for spiking experiments. The MCF7 adenocarcinoma cells were cultured in 1:1 Dulbecco’s modified Eagle medium (DMEM Glutamax) (GIBCO-BRL Co, MD USA) supplemented with 10% fetal bovine serum (FBS) (GIBCO-BRL), 16 ng/ml insulin, and 50 mg/ml penicillin/streptomycin (GIBCO-BRL). MDA-MB-231 cells were cultured in DMEM, supplemented with 10% FBS and 50 mg/ml penicillin/streptomycin. SKBR3 breast cancer cells were cultured in RPMI supplemented with 10% FBS. Hela were cultured in 1:1 Dulbecco’s modified Eagle medium (DMEM Glutamax) supplemented with 10% FBS and 50 mg/ml penicillin/streptomycin. Cells were maintained in a humidified atmosphere of 5% CO_2_ in the air. Sub-cultivation was performed with 0.25% trypsin and 5 mM EDTA (GIBCO-BRL). All experiments were performed during the logarithmic growth phase.

### Double staining experiments and confocal laser scanning microscopy

The presence of CK-positive cells in PBMCs’ cytospin was investigated using the mouse A45-B/B3 (detecting CK8, CK18, and CK19) (Micromet Munich, Germany) antibody and anti-CD45 (common leukocyte antigen) (Santa Cruz, Santa Cruz, CA, USA) in order to exclude possible ectopic expression of cytokeratin on hematopoietic cells. The cyto-morphological criteria proposed by Meng et al. [40] (i.e., high nuclear/cytoplasmic ratio, larger cells than white blood cells, etc.) were used in order to characterize a CK-positive cell as a CTC.

Fixed cells with acetone/methanol (9:1 *v*/*v*) were stained with A45-B/B3 antibody for 1 h, followed by Alexa488 anti-mouse for 45 min. Afterwards, CD45 anti-rabbit was used for 1 h. Consequently, the samples were stained with the corresponding Alexa555 anti-rabbit fluorochrome. Finally, cells were stained with DAPI (Invitrogen, Carlsbad, CA, USA) conjugated with antifade and analyzed with confocal laser scanning microscopy.

### Triple immunofluorescence

Triple immunofluorescence was performed to cytospins’ preparations from all patients harboring CK (+)/CD45 (−) CTCs. For the CK/CXCR4/JUNB staining, cells were fixed with 3% paraformaldehyde (PFA) for 30 min at room temperature (RT). Permeabilization was achieved with 0.5% Triton X-100 for 10 min at RT. After blocking with PBS supplemented with 10% (*v*/*v*) FBS for 1 h, cells were incubated with A45-B/B3 for 1 h and then with Alexa 488 for 45 min. Consequently, slides were incubated with JUNB antibody conjugated with Alexa647 (Santa Cruz, CA, USA) for 1 h. CXCR4 anti-rabbit antibody (ABCAM, Cambridge, MA USA) was added to the samples for 1 h, followed by the corresponding Alexa555 anti-rabbit (Molecular Probes, Invitrogen, Carlsbad, CA, USA) for 45 min. Finally, cells were stained with DAPI conjugated with antifade.

For the triple staining with the CK/PRDX1 (Santa Cruz, USA)/TYROBP (Abcam) or the CK/NFYA (Abcam)**/**YWHAB (Santa Cruz), cells were initially incubated with acetone/methanol (9:1) solution for 10 min and, then, washed 3 times with PBS. Blocking solution with 10% (*v*/*v*) FBS in PBS for 1 h was used to eliminate non-specific binding. Consequently, slides were incubated with the corresponding primary and secondary antibodies. Zenon technology (FITC-conjugated IGg1 antibody) (Molecular Probes, Invitrogen) was used for CK detection with the A45-B/B3 antibody. Zenon antibodies were prepared within 30 min before use. Cells were also stained with DAPI conjugated with antifade.

Positive and negative controls were used in each experiment (Additional file 1: Figure S1, Additional file 2: Figure S2), using breast cancer cell lines’ cytospins by omitting one of the first antibodies. Therefore, each experiment included three different negative controls and one positive for all the antibodies.

Slides were, then, analyzed with confocal laser scanning microscopy, while the quantification of the protein expression was performed using the software platform of the ARIOL microscope. The platform automatically measured the intensity per pixel of each fluorochrome in every distinct isolated CTC.

### Statistical analysis of the clinical data

All statistical tests were performed at the 5% level of significance. SPSS version 20 (SPSS Inc., Chicago, IL) statistical software was used for the analysis. Overall survival (OS) was defined as the time from treatment initiation, until death from any cause. Progression-free survival (PFS) was defined from the enrolment to the study until disease relapse or death whatever occurred first. Kaplan-Meier curves and Cox regression analysis for PFS and OS were compared using the log-rank test to provide a univariate and multivariate assessment of the prognostic value of selected clinical risk factors.

### Immunohistochemistry: JUNB staining in tissue samples

Eleven primary and four metastatic tumors were available for JUNB staining from the same cohort of patients. One patient had available primary breast tumor and pleural metastasis. Three-micrometer-thick formalin-fixed paraffin-embedded (FFPE) tissue sections on charged glass slides were deparaffinized in xylene and rehydrated in a graded series of ethanols. Epitope retrieval was heat-induced in a vegetable steamer treated for 45 min in a solution buffered with citric acid (pH 6). Endogenous peroxidase was blocked by applying UltraVision Hydrogen Peroxide Block (Thermo Scientific, Waltham, MA) for 15 min. Nonspecific protein-binding sites were blocked with UltraVision Protein Block (Thermo Scientific, Waltham, MA) for 5 min. Sections were stained with Jun B mouse monoclonal antibody clone C-11 (1:50) (Santa Cruz, CA, USA) for 1 h at room temperature. Immunodetection was performed using UltraVision Quanto Detection System HRP Polymer DAB (Thermo Scientific, Waltham, MA, USA) according to the manufacturer’s instructions. 3,3′Diaminobenzidine Quanto Chromogen (Thermo Scientific, Waltham, MA) was used as chromogen. Slides were counterstained with hematoxylin.

Samples’ histoscore (*H*-score) was calculated by a semi-quantitative assessment of both the intensity of staining (graded as 0, no-staining; 1, weak; 2, medium; or 3, strong) and the percentage of positive cells. The percentage of cells at each staining intensity level was calculated, and an *H*-score was assigned summing the individual *H*-scores for each intensity level using the following formula: [1 × (% cells 1+) + 2 × (% cells 2+) + 3 × (% cells 3+)]. The range of possible scores was from 0 to 300.

## Results

### Bioinformatics results and functional enrichment evaluation

**Fig. 1:**
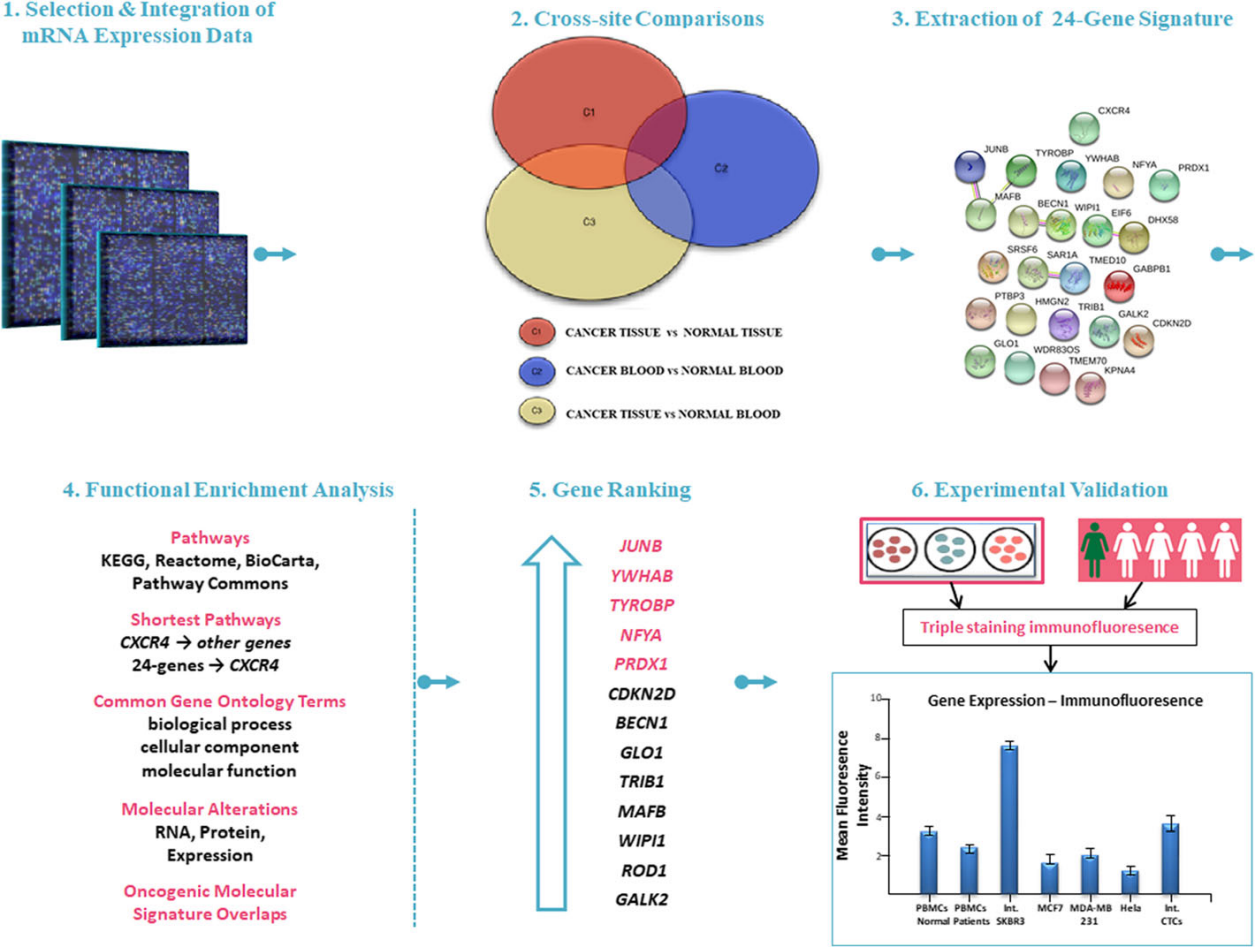
Bioinformatics and functional enrichment analysis for biomarker discovery. Twenty-four genes were obtained from a data-driven computational procedure (data integration, cross-site comparisons) and were further explored by using functional enrichment analysis. CXCR4 plays a central role in this analysis. Based on four databases (G2SBC, WebGestalt, GSEA, and KEGG), significant biological information was validated and five genes of the 24-signature were prioritized according to their direct or indirect association with the CXCR4 pathway. These six molecules (CXCR4, *JUNB*, *YWHAB*, *TYROBP*, *NFYA*, and *PRDX1*) were experimentally evaluated in biological samples

Based on our previous work [22], we performed a cross-platform comparative study of integrated public datasets, which revealed gene signatures exhibiting differentially overexpression patterns. In particular, the integrated datasets were of different “origin” (peripheral blood and tissue) and disease status (healthy and cancerous), and three different comparisons were performed (Fig. 1): cancer versus healthy tissue samples (Comparison 1, C1), cancer versus healthy blood samples (C2), and cancer tissue versus healthy blood samples (C3). These comparative analyses aimed to reveal persisting differences in the expression profile of certain genes in healthy and cancer status and possibly identify a limited set of candidate CTC biomarkers. For the C1 comparison, a total of 3725 genes were identified, exhibiting significant differential overexpression in cancer cells over control tissue samples. In the same way, 79 overexpressed genes were derived from the C2 comparison in cancerous peripheral blood (PB) samples and 245 overexpressed genes were extracted from the C3 comparison in cancer tissue samples over control PB samples. Based on our hypothesis, the peripheral blood carries information regarding both the primary and metastasis, as well as other cancer-induced alterations. We considered the intersection (C1∩C2∩C3) of the three gene signatures as a target of our biological assessment, since it includes only genes overexpressed in PB, related to cancer-associated factors, eliminating genes overexpressed in normal PB. This common set includes 24 genes, and CXCR4 plays a central role. A functional enrichment evaluation was further performed. Functional enrichment evaluation is generally recognized as a secondary analysis on large gene sets, resulted from high-throughput genomic methods. This step allowed the validation of the gene signature status, based on the biological significance of the 24 extracted genes. We further analyzed each of the 24 genes for their “intrinsic” properties and the 24-gene signature as whole according to their direct or indirect link with (i) *CXCR4* pathway and (ii) *CXCR4* biological features. Relevant biological information was extracted from four databases as follows: (a) pathways were acquired from the G2SBC (Genes-to-Systems Breast Cancer) [41], KEGG (Kyoto Encyclopedia of Genes and Genomes) [42], Gene Set Enrichment Analysis (GSEA) [43], and WebGestalt (WEB-based GEne SeT AnaLysis Toolkit) [44] in the category of “Pathway Commons” (PC); (b) molecular alterations in breast cancer, and the shortest pathways from *CXCR4* to other genes, were delivered from G2SBC; (c) gene ontology (GO) terms were yielded from G2SBC and GSEA; and (d) oncogenic molecular signature overlaps were computed from GSEA. Only genes participating in enriched biological terms at the *P* ≤ 0.05 level, after multiple test correction [38], were considered as candidate genes and, if they shared multiple biological functions with CXCR4, were prioritized for further analysis in biological samples. Five genes, named JUNB, YWHAB, TYROBP, NFYA, and PRDX1, were evaluated as highly ranked genes, which could be tested in samples from mBC patients and cell lines (Fig. 1).

### Expression pattern of CXCR4 and JUNB in patients’ CTCs

**Fig. 2:**
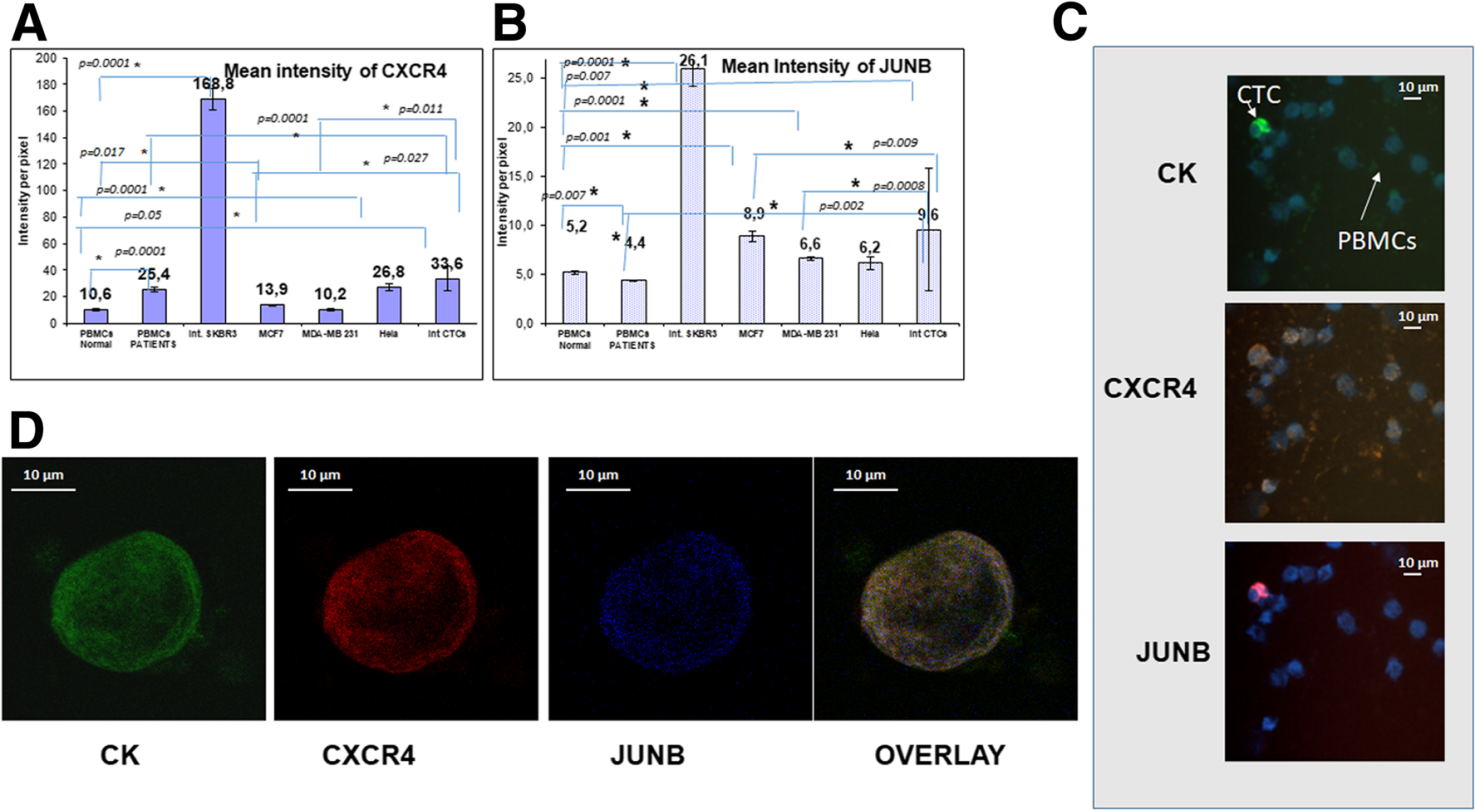
Expression of cytokeratin, CXCR4, and JUNB in CTCs isolated from breast cancer patients. **a** Quantification of CXCR4 expression (intensity per pixel) in breast cancer cell lines, Hela cells, PBMCs, and patients’ CTCs, using ARIOL system automate software. **b** Quantification of JUNB expression (intensity per pixel) in breast cancer cell lines, in Hela cells, PBMCs, and patients’ CTCs, using ARIOL system automate software. **c** Cytospins obtained from metastatic breast cancer patients were triple stained with CK (green), CXCR4 (orange), and JUNB (red) antibodies plus DAPI (blue) and analyzed with ARIOL system (magnification X40). **d** Representative images from confocal laser scanning microscopy. Patients’ samples were triple stained with CK (green), CXCR4 (red), and JUNB (blue) antibodies plus DAPI (not shown due to the absence of corresponding laser) magnification (× 60)

Quantification of the mean intensity per pixel of each fluorochrome by the ARIOL system provided an expression pattern of every investigated protein in all the examined breast cancer cells lines from different subtypes (Luminal: MCF7, HER-positive: SKBR3, basal-like: MDA-MB 231). In addition, the expression of each protein in normal donors’ PBMCs was quantified and compared to patients’ monocytes. Hela cells were used in the analysis, because they were suggested by the manufacturers as positive controls for some of the antibodies (Fig. 2).

In the breast cancer cell lines MCF7 and SKBR3, the intensity of CXCR4 was statistically increased (*p* = 0.017 and *p* = 0.0001 respectively) compared to normal PBMCs (Fig. 2a). In addition, the mean intensity of CXCR4 in normal donors’ PBMCs was 10.62 ± 1.01, while in patients’ blood, the mean intensity was enhanced (25.4 ± 1.59; *p* = 0.0001; Table 2) compared to normal PBMCs. Furthermore, the mean intensity of isolated CTCs in the patients was 33.57 ± 9.25 which was statistically higher compared to normal donors’ PBMCs (*p* = 0.05) and to patients’ PBMCs (*p* = 0.0001). Moreover, the expression of CXCR4 was higher in CTCs compared to MCF7 (*p* = 0.0027) and to MDA-MB 231 (*p* = 0.011) (Table 2, Fig. 2a). Setting as the threshold value the mean intensity of CXCR4 in normal donors’ PBMCs, 90% (18 out of 20) of the patients with detectable tumor cells in their blood harvested CTCs positive for CXCR4. In addition, 82.61% of the total isolated CTCs have an intensity higher than the threshold and considered positive for CXCR4.

### JUNB expression in CTCs

JUNB intensity was statistically higher in all breast cancer cell lines (SKBR3: *p* = 0.0001, MCF7: *p* = 0.001, MDA-MB231: *p* = 0.0001) compared to normal donors’ PBMCs (Table 2, Fig. 2b). In addition, the intensity of JUNB in isolated patients’ CTCs was statistically higher compared to normal (*p* = 0.007) and patients’ (*p* = 0.002) PBMCs. As it is shown in Fig. 2c, d, JUNB expression in CTCs was enhanced compared to patients’ PBMCs, providing a potential biomarker to distinct cancer cells in patients’ blood.

Furthermore, the intensity of JUNB was increased compared to MCF7 (*p* = 0.009) and MDA-MB231 (*p* = 0.0008) cells. Using as threshold value the mean intensity of JUNB in normal PBMCs, 65% (13 out of 20) of the patients with detectable tumor cells in their blood harbored CTCs positive for JUNB. In addition, 78.57% of the total isolated CTCs were JUNB-positive.

### Expression pattern of TYROBP and PRDX1 in breast cancer cell lines and in patients’ CTCs

**Fig. 3:**
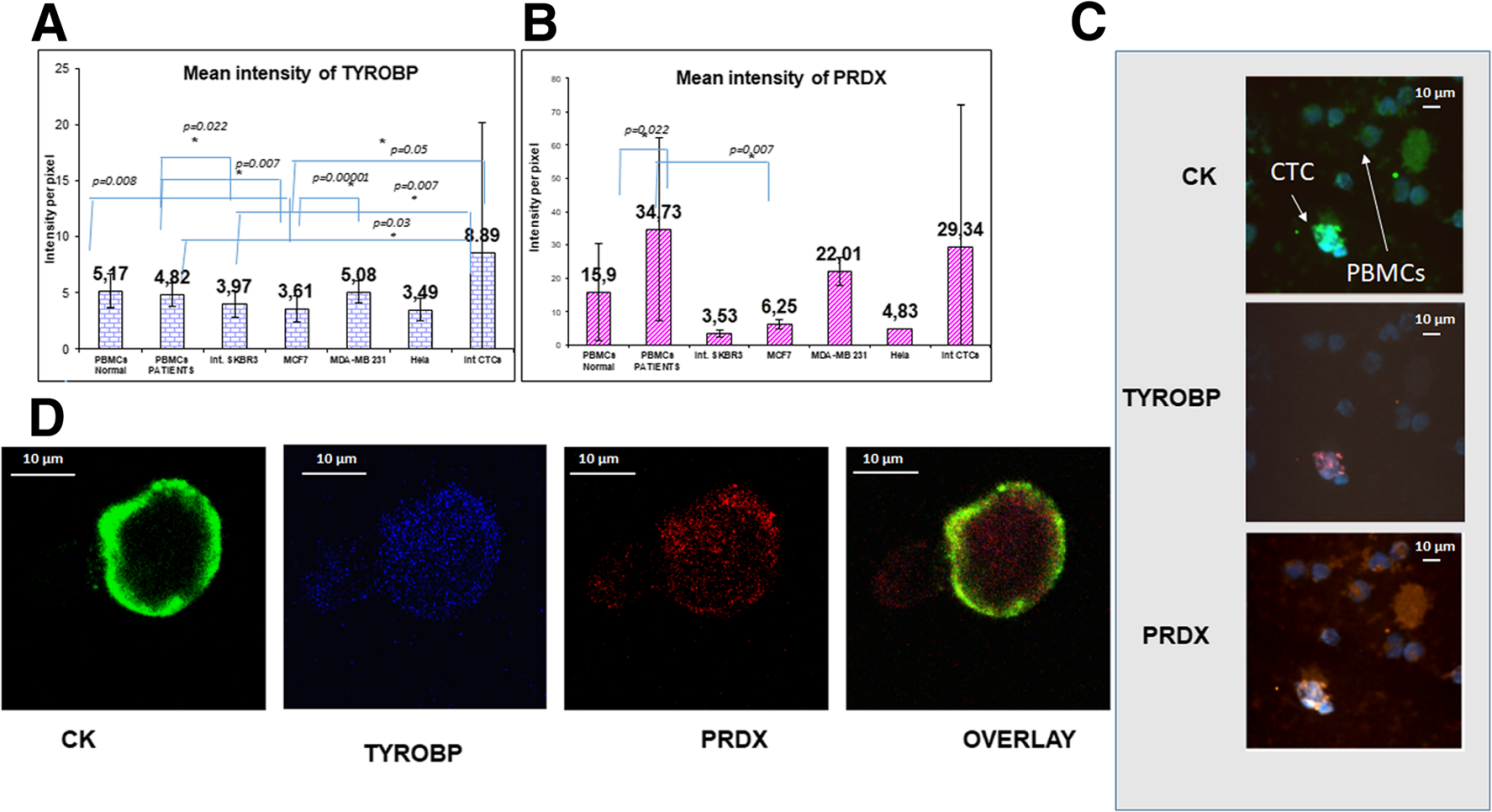
Expression of cytokeratin, TYROBP, and PRDX1 in CTCs isolated from breast cancer patients. **a** Quantification of TYROBP expression (intensity per pixel) in breast cancer cell lines, Hela cells, PBMCs, and patients’ CTCs, using ARIOL system automate software. **b** Quantification of PRDX1 expression (intensity per pixel) in breast cancer cell lines, in Hela cells, in PBMCs, and in patients’ CTCs, using ARIOL system automate software. **c** Cytospins obtained from metastatic breast cancer patients were triple stained with CK (green), PRDX1(orange), and TYROBP (red) antibodies plus DAPI (blue) and analyzed with ARIOL system (magnification × 40). **d** Representative images from confocal laser scanning microscopy. Patients’ samples were triple stained with CK (green), TYROBP (blue), and PRDX1 (red) antibodies plus DAPI (not shown due to the absence of corresponding laser) and analyzed with confocal laser scanning microscopy (magnification × 60)

Quantification of TYROBP expression in breast cancer cell lines and in PBMCs from normal donors and patients revealed that this protein was downregulated in MCF7 (*p* = 0.008) cells compared to normal PBMCs. In addition, a significant difference was observed between the mean intensity of TYROBP in patients’ PBMCs and breast cancer cell lines [SKBR3 (*p* = 0.022), MCF7 (*p* = 0.007)] (Table 2 and Fig. 3a).

The mean intensity of TYPORB expression in patients’ CTCs (8.89 ± 3.53) was higher than that of breast cancer cell lines [MCF 7 (3.61 ± 0.1, *p* = 0.05), SKBR3 (3.97 ± 0.13, p = 0.007), MDA-MB231 (5.08 ± 0.09, *p* = 0.11)]. It was also higher compared to normal (5.17 ± 0.67, *p* = 0.322) and to patients’ (4.82 ± 0.21, *p* = 0.03) PBMCs.

Using as threshold value the intensity of TYROBP in normal PBMCs, 64.3% of the isolated CTCs were positive for TYROBP. In addition, 75% (12 out of 16) of the patients with detectable tumor cells in their blood harvested TYROBP-positive CTCs.

### PRDX1 expression in CTCs

PRDX1 was also quantified in the same cohort of patients (Table 2). The results revealed that PRDX1 expression was higher in MDA-MB231 cells (22.0 ± 1.64, *p* = 0.12) compared to normal PBMCs (15.9 ± 3.62). In addition, the mean PRDX1 expression in CTCs (29.34 ± 24.6) was numerically, but not statistically higher than that observed in breast cancer cell lines and in the normal PBMCs (15.9 ± 3.62) (Table 2, Fig. 3b).

Using as threshold value the mean intensity of PRDX1 in normal PBMCs, 20% of the isolated CTCs were positive for PRDX1. In addition, only 12.5% (2 out of 16) of the patients with detectable tumor cells in their blood had detectable PRDX1-positive CTCs.

### Expression pattern of NFYA and YWHAB in breast cancer cell lines and in patients’ CTCs

**Fig. 4:**
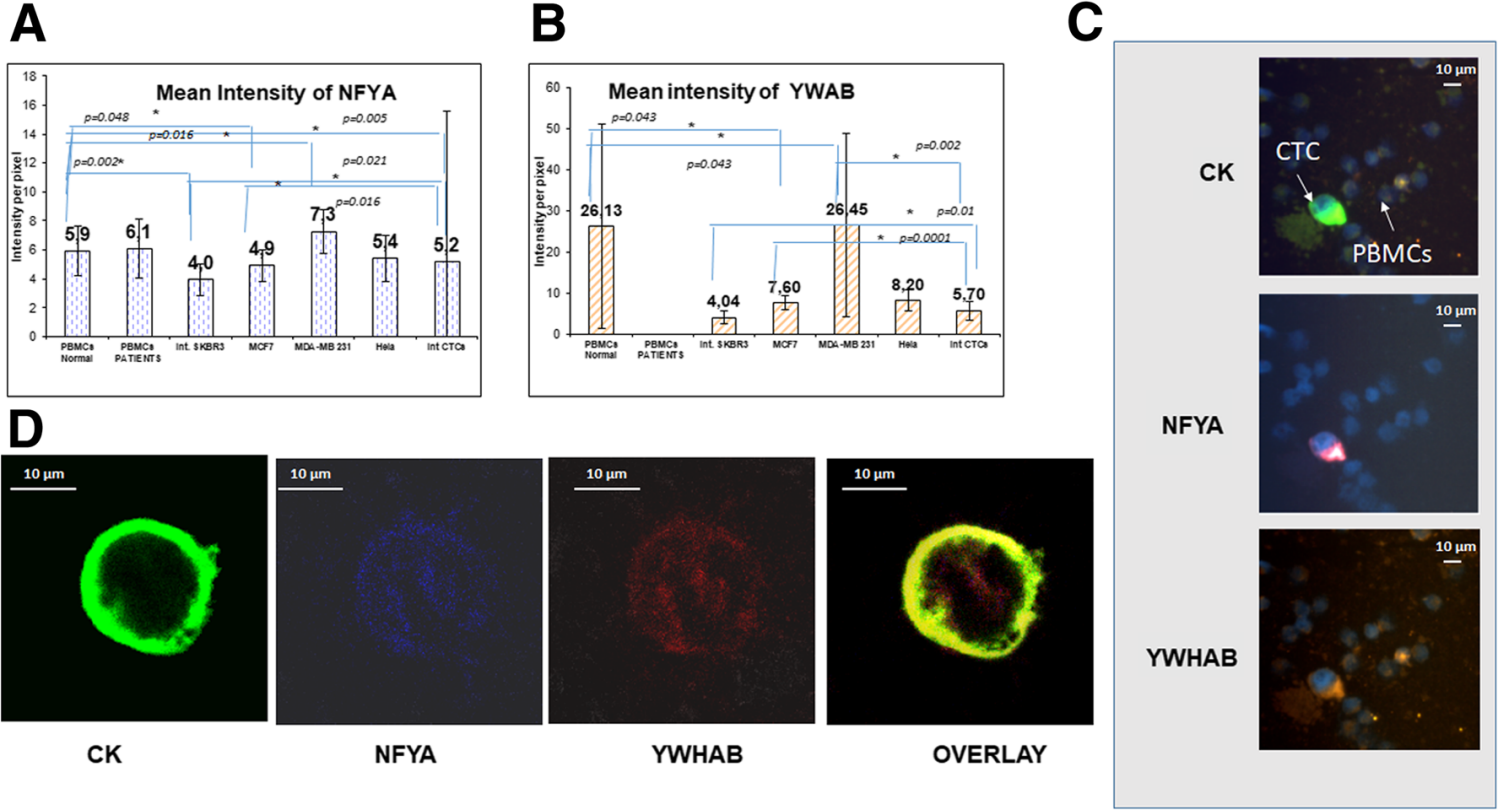
Expression of cytokeratin, NFYA, and YWHAB in CTCs isolated from breast cancer patients. **a** Quantification of NFYA expression (intensity per pixel) in breast cancer cell lines, Hela cells, PBMCs, and patients’ CTCs, using ARIOL system automate software. **b** Quantification of YWHAB expression (intensity per pixel) in breast cancer cell lines, in Hela cells, in PBMCs, and in patients’ CTCs, using ARIOL system automate software. **c** Cytospins obtained from metastatic breast cancer patients were triple stained with CK (green), YWHAB (orange), and NFYA (red) antibodies plus DAPI (blue) and analyzed with ARIOL system (magnification × 40). **d** Representative images from confocal laser scanning microscopy. Patients’ samples were triple stained with CK (green), NFYA (blue), and YWHAB (red) antibodies plus DAPI (not shown due to the absence of the corresponding laser) and analyzed with confocal laser scanning microscopy (magnification × 60)

Evaluation of mean intensity of NFYA revealed that the highest expression among the breast cancer cell lines was observed in MDA-MB 231 [(Table 2), 7.26 ± 0.15)] which was statistically different compared to normal donors’ PBMCs (5.9 ± 0.52, *p* = 0.016). The intensity of NFYA expression in patients’ CTCs (5.20 ± 1.36) was higher compared to MCF7 (4.93 ± 0.016) and SKBR3 (3.95 ± 0.021) cells, but it was lower than in normal PBMCs (5.9 ± 0.52, *p* = 0.005; Fig. 4a).

Using as a threshold the intensity of NFYA expression in normal donors’ PBMCs, only 6.45% of the whole number of isolated CTCs were positive for this protein. In addition, only 14.3% (2 out of 14) of the patients with detectable CK-positive cells were considered positive for NFYA.

### YWHAB expression in CTCs

The intensity of YWHAB was also evaluated in breast cancer cell lines and in patients’ CTCs. The mean intensity of YWHAB expression in normal donors’ PBMCs (26.13 ± 10.10) was higher than that observed in MCF7 (7.60 ± 0.12, *p* = 0.043) and SKBR3 (4.04 ± 0.15, *p* = 0.058) cell lines. In contrast, MDA-MB231 (26.45 ± 3.52, *p* = 0.043) cells revealed significant higher expression compared to normal PBMCs.

In patients’ CTCs, the mean intensity of YWHAB expression was 5.70 ± 0.28, which was significantly higher than the intensity observed in SKBR3 (4.04 ± 0.15, *p* = 0.01) cells, whereas it was lower compared to MCF7 (7.6 ± 0.12, *p* = 0.0001) and MDA-MB231 (26.45 ± 3.52, *p* = 0.002) cells. The mean intensity of YWHAB expression in CTCs was numerically but not significantly lower than in normal PBMCs (*p* = 0.07; Fig. 4b). None of the patients could be considered positive for YWHAB, taking as threshold value the normal donors’ PBMCS mean intensity.

### Clinical impact of the evaluated molecules

**Fig. 5:**
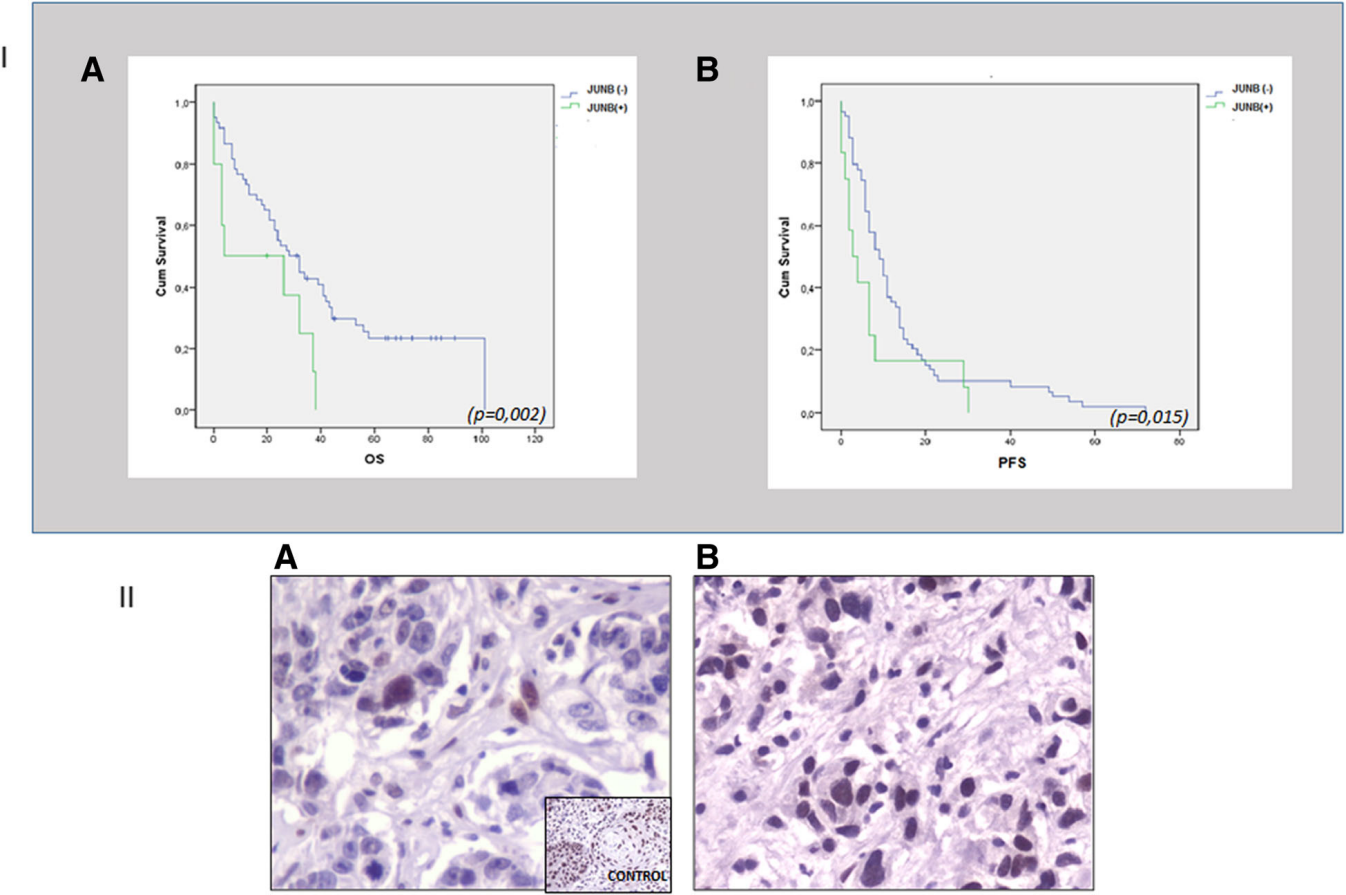
**I** (A) Overall survival in patients with JUNB-positive CTCs compared to JUNB-negative patients (*p* = 0.02, Kaplan-Meier analysis). In the group of patients harboring JUNB-positive CTCs, 90% died during follow-up period vs 75% of deaths in the cohort of patients without JUNB expression. **I** (B) Kaplan-Meier analysis revealed that the progression-free survival in patients harboring JUNB-positive CTCs was statistically lower compared to patients without JUNB expression (*p* = 0.015). Patients without JUNB-positive CTCs experienced longer PFS vs JUNB-negative patients [3.5 (range 0–30) vs 9 (range 0–72) months]. **II** (A) JUNB staining in primary tumor obtained from BC patient. JUNB-positive tumor cells (brown) were very rare in the sample. Inner frame is shown control sample from a squamous cell carcinoma (magnification × 40). **II** (B) JUNB staining in metastatic tumor obtained from mBC patient. The majority of the tumor cells were positive for JUNB (magnification × 40)

Analysis of the clinical data of all the examined patients (100) revealed that after a median follow-up period of 24 months (range 1–101), 60 patients died due to disease progression. The median overall survival (OS) was significantly lower, in the group of patients harboring JUNB-positive CTCs (HR = 2.308, *p* = 0.026, Cox regression and *p* = 0.02: Kaplan-Meier analysis), compared to patients without JUNB-expression in their CTCs [(17 months (range 0–38) vs 24.5 months (range 0–101); Fig. 5 (A)]. Particularly in the group of patients with JUNB-positive CTCs, 90% died during the follow-up period (9 out of 10 with available follow-up data) while in the cohort of patients without JUNB expression, 75% (45 out of 90) died during the follow-up period. Similarly, the median progression-free survival (PFS) was significantly lower in patients harboring JUNB-positive CTCs compared to patients without JUNB-positive CTCs [3.5 months (range 0–30) vs 9 months (range 0–72), *p* = 0.015, Kaplan-Meier analysis] (Fig. 5 (B)). Interestingly, the presence of CK/CD45 cells in these patients did not correlate with clinical outcome. Furthermore, statistical analysis of the rest of the examined proteins did not lead to clinical correlation with OS or PFS. Multivariate analysis revealed that JUNB expression in CTCs is an independent prognostic factor (*p* = 0.016, HR 2.2484) for OS in breast cancer patients.

Further analysis of the other clinicopathological characteristics revealed that reduced OS was significantly associated with performance status (PS) (*p* = 0.001, HR = 2.391, Cox regression).

### JUNB expression in primary tumors

Eleven primary tumors from the same cohort of patients were screened for JUNB expression. Eight of them were negative for JUNB. Mostly all of the blood samples from these patients were also negative for CTCs. Only one patient in this group harbored JUNB-positive CTCs.

Three primary tumor samples revealed low expression of JUNB (*H*-scores 5, 6, and 9). One of them had also JUNB-positive CTCs.

On the other hand, all the examined samples (four) from metastatic tumors were positive for JUNB and the *H*-scores were very high (20, 120, 105, and 140). Three of these patients harbored also JUNB-positive CTCs in their blood.

It is interesting that in a patient with available primary tumor and pleura metastasis, the sample from the primary tumor was completely negative for JUNB expression (*H*-score 0), while in metastasis the *H*-score was extreme (120). This patient harbored also JUNB-positive CTCs (Table 3).

**Table 3:** JUNB expression in primary and metastatic tumors

Patient	Material	JUN B IHC EVAL - FFPE tissues			JUNB positive (+) CTCs
		% (0–100)	Intensity (0–3)	H-score* (0–300)	
1	Primary tumor	0	0	*0*	–
2	Primary tumor	0	0	*0*	+
3	Primary tumor	3	2	*6*	+
4	Primary tumor	5	1	*5*	Negative for CTCs
5	Primary tumor	0	0	*0*	Negative for CTCs
6	Primary tumor	0	0	*0*	Negative for CTCs
7	Primary tumor	0	0	*0*	Negative for CTCs
8	Primary tumor	9	1	*9*	–
9	Primary tumor	0	0	*0*	Negative for CTCs
10	Primary tumor	0	0	*0*	Negative for CTCs
11	Primary tumor	0	0	*0*	+
	Metastasis	40	3	*120*	
12	Metastasis	10	2	*20*	Negative for CTCs
13	Metastasis	35	3	*105*	+
14	Metastasis	40 &20	2&3	*140*	+

*Histoscore (H-score) was calculated by a semi-quantitative assessment of both the intensity of staining (graded as: 0: no-staining; 1: weak; 2: medium; or 3: strong) and the percentage of positive cells. The percentage of cells at each staining intensity level was calculated, and an H-score was assigned summming the individual H-scores for each intensity level using the following formula: [1 × (% cells 1+) + 2 × (% cells 2+) + 3 × (% cells 3+)]. The range of possible scores was from 0 to 300

## Discussion

It is widely accepted that the detection of CTCs is a valuable parameter for the assessment of prognosis in cancer patients. CTCs can also be an important therapeutic target since in a randomized phase II trial it was observed that in patients with HER2-negative early breast cancer targeting HER2-positive CTCs with trastuzumab after the completion of adjuvant chemotherapy resulted in a significant improvement of patients’ DFS [45]. However, just the enumeration of CTCs is not sufficient for defining prognosis in cancer patients. Conversely, their characterization, which could be based on the expression of different proteins, is critical, since it allows the identification of CTCs undergoing EMT or expressing stem cell markers. In addition, it has been recently reported that certain CTCs’ phenotypes can be related to metastatic procedure [1, 2]. Therefore, it is obvious that there is an unmet need for the development of new biomarkers for their detection and characterization. Bioinformatics analysis, based on gene expression databases, could help to this end.

Current study investigated whether bioinformatics analysis using public databases could define putative biomarkers with clinical relevance in CTCs, isolated from metastatic breast cancer patients. The presented bioinformatics data revealed that 6 genes could be potentially overexpressed in CTCs derived from breast cancer patients. All these genes are related to different CXCR4 signal transduction pathways. CXCR4 is a chemokine receptor that binds CXCL12. It has been shown that CXCR4 is upregulated in a number of tumors, such as breast, melanoma, ovarian, and prostate cancer, whereas its expression is low in normal tissues [46, 47]. It is also interesting to note that CXCR4 is induced by angiogenic factors, such as VEGF, and previous studies from our group had shown that VEGF and VEGFR2 were upregulated in CTCs derived from metastatic breast cancer patients [39]. Interestingly in the present study, 90% of the patients with detectable CTCs in their blood harbored CTCs with CXCR4-positive phenotype. Furthermore, quantification of this expression (intensity per pixel) revealed that CXCR4 is significantly upregulated in patients’ CTCs compared to MCF7 (*p* = 0.027) and MDA-MB231 (*p* = 0.011) cells and in comparison to normal PBMCs (*p* = 0.05). This observation strongly suggests that CXCR4 receptor is implicated to the metastatic process. These results confirm the conclusions of our bioinformatics analysis, and they are in agreement with previous studies regarding the CXCR4 mRNA expression in CTCs from breast cancer patients [24, 48, 49]. CXCR4 expression in these studies was associated with the presence of > 3 positive lymph nodes and with EMT markers such as Vimentin and Snail [24] whereas Mego et al. [48] reported that CXCR4 expression was related to CTCs exhibiting epithelial markers. In addition, CXCR4 was upregulated in CTCs derived from different tumor types such as small cell lung cancer, and this expression was associated with poorer PFS [50]. However, in the current study although 82.6% of the whole number of detected CTCs were CXCR4-positive, this detection was not associated with the clinical outcome. Conversely, patients with JUNB expression in their CTCs revealed poorer OS (*p* = 0.002) and PFS (*p* = 0.015) compared to patients without this phenotype. Moreover, multivariate analysis showed that the detection of JUNB in CTCs could be emerged as an independent prognostic factor, associated with poor clinical outcome in terms of OS (*p* = 0.016). This clinical relevance of JUNB-positive CTCs raises the question of its significance as potential therapeutic target for the elimination of these cells. However, analysis of the different breast cancer patients’ subtypes (hormone receptor-positive, HER2-positive, triple negative) separately did not reveal statistical differences.

The data of the current study are in line with previous studies reporting the role of JUNB in cancer progression, such as Hodgkin’s disease and anaplastic large cell lymphomas [51, 52]. Moreover, it has been shown that JUNB is induced by ALK-NPM, participating the mTOR pathway [53] and is required for cell cycle re-entry, after quiescence, and it cooperates with c-jun for the development of fibrosarcoma [54]. In addition, there are some recent data indicating that JUNB is implicated in the earliest events of development of resistance to kinase inhibitors in breast cancer [30]. However in the current study, JUNB overexpression could not be attributed to the treatment, because the samples were obtained before the initiation of first-line treatment. Combining all these data with our findings strongly supports that JUNB plays a critical role in cancer metastasis. This assumption was reinforced by the analysis of 11 primary tumors from the same group of patients, showing that only three of them revealed low JUNB expression, while the rest were completely negative. However, all the metastatic tumors (four) were strongly positive for JUNB. Furthermore, in one patient with available primary tumor and metastasis, JUNB was dramatically increased (from *H*-score 0 to *H*-score 120) in metastatic tissue, denoting the critical role of this molecule in cancer progression.

Our data also indicate an increased intensity of PRDX1 expression in patients’ CTCs compared to normal PBMCs, but this difference was not statistically significant. This observation is in line with previous studies demonstrating that PRDX is increased in the most aggressive [triple negative (TN)] subtype of breast cancer patients [55]. It is interesting that previous studies have shown that PRDX provide a protective role in cancer cells regarding doxorubicin-induced toxicity and they are associated with overall mortality in breast cancer patients [33, 35].

The intensity of TYROBP expression was also increased in CTCs compared to normal PBMCs and MCF7 cells, implying an upregulation of this protein in aggressive metastatic cancer cells. The strong statistical difference of TYROBP intensity between MCF7 and MDA-MB 231 (*p* = 0.00001) reinforced this assumption.

Conversely, although bioinformatics analysis revealed that NFYA and YWHAB genes’ expression could be upregulated in CTCs, our results did not confirm this assumption. This could be attributed to post-transcriptional or post-translational modifications that prohibit the upregulation of protein levels of NFYA and YWHAB.

## Conclusions

Bioinformatics analysis could be a useful tool for the identification of new biomarkers and therapeutic targets. Experimental approach to this analysis confirmed the overexpression of CXCR4, JUNB, and TYROBP. In addition, the presence of JUNB-positive CTCs emerged as an independent prognostic factor for OS in breast cancer patients.

## Additional files

Additional file 1: **Figure S1.** Positive and negative controls of CK/CD45 and CK/JUNB/CXCR4 stainings. (IA): Positive controls for CK/CD45 staining: Cytospins with SKBR3 cells spiked in normal donor’s PBMCs were stained with CK (green) anti-mouse, Alexa 488 anti-mouse, CD45 (blue) anti-rabbit, and Alexa 633 anti-rabbit antibodies. (IB): Negative controls for CK/CD45 staining: Cytospins were stained with all the above antibodies except the primary CK anti-mouse antibody. (IIA) Positive controls for CK/JUNB/CXCR4 staining: Cytospins with SKBR3 cells were stained with CK (green), JUNB (blue), CXCR4 (red) antibodies, and the corresponding fluorochromes. (IB) Negative controls for CK/JUNB/CXCR4 staining: Cytospins were stained with all the corresponding antibodies except the primary CXCR4 antibody. (IIC) Negative controls for CK/JUNB/CXCR4 staining: Cells were stained with all the corresponding antibodies except the primary JUNB antibody. (TIF 4960 kb)

Additional file 2: **Figure S2.** (IA) Positive controls for CK/TYROBP/PRDX1 staining: MDA-MB 231 cells were stained with CK (green), TYROBP (blue), PRDX1 (red) antibodies, and the corresponding fluorochromes. (IB) Negative controls for CK/TYROBP/PRDX1 staining: Cells were stained with all the corresponding antibodies except the primary TYROBP antibody. (IC) Negative controls for CK/TYROBP/PRDX1 staining: Cells were stained with all the corresponding antibodies except the primary PRDX1 antibody. (IIA) Positive controls for CK/NFYA/YWHAB staining: MDA-MB 231 cells were stained with CK (green), NFYA (blue), YWHAB (red) antibodies, and the corresponding fluorochromes. (IIB) Negative controls for CK/NFYA/YWHAB staining: Cells were stained with all the corresponding antibodies except the primary NFYA antibody. (IIC) Negative controls for CK/NFYA/YWHAB staining: Cells were stained with all the corresponding antibodies except the primary YWHAB antibody. (TIF 4741 kb)

## Data Availability

The datasets used and/or analyzed during the current study are available from the corresponding author on reasonable request.

## Abbreviations

BC: Breast cancer
CK: Cytokeratin
CTCs: Circulating tumor cells
CXCR4: C-X-C chemokine receptor type 4
EMT: Epithelial-mesenchymal transition
NFYA: Nuclear transcription factor Y subunit alpha
OS: Overall survival
PBMCs: Peripheral blood mononuclear cells
PFS: Progression-free survival
PRDX: Peroxiredoxin
TYROBP (DAP12): Tyrosine kinase-binding protein
