# Supplementary figures and images for: The prognostic value of JUNB-positive CTCs in metastatic breast cancer: from bioinformatics to phenotypic characterization
Source: Breast Cancer Res. 2019 Aug 1;21:86. doi: 10.1186/s13058-019-1166-4 (PMC6676640; doi:10.1186/s13058-019-1166-4)

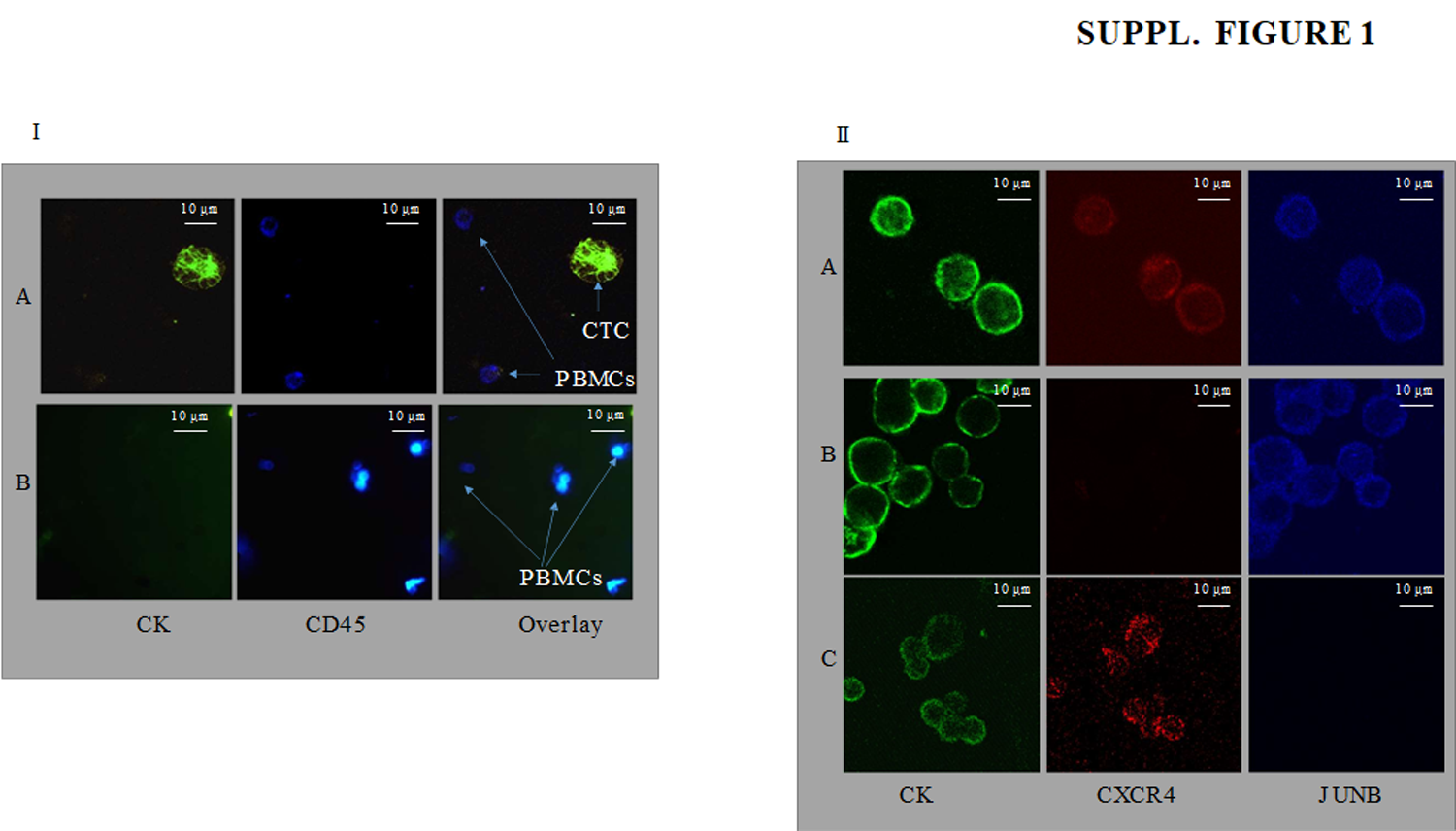

Supplement: Supplementary file 1 — Figure S1. Positive and negative controls of CK/CD45 and CK/JUNB/CXCR4 stainings. (IA): Positive controls for CK/CD45 staining: Cytospins with SKBR3 cells spiked in normal donor’s PBMCs were stained with CK (green) anti-mouse, Alexa 488 anti-mouse, CD45 (blue) anti-rabbit, and Alexa 633 anti-rabbit antibodies. (IB): Negative controls for CK/CD45 staining: Cytospins were stained with all the above antibodies except the primary CK anti-mouse antibody. (IIA) Positive controls for CK/JUNB/CXCR4 staining: Cytospins with SKBR3 cells were stained with CK (green), JUNB (blue), CXCR4 (red) antibodies, and the corresponding fluorochromes. (IB) Negative controls for CK/JUNB/CXCR4 staining: Cytospins were stained with all the corresponding antibodies except the primary CXCR4 antibody. (IIC) Negative controls for CK/JUNB/CXCR4 staining: Cells were stained with all the corresponding antibodies except the primary JUNB antibody. (TIF 4960 kb) [file 13058_2019_1166_MOESM1_ESM.tif]

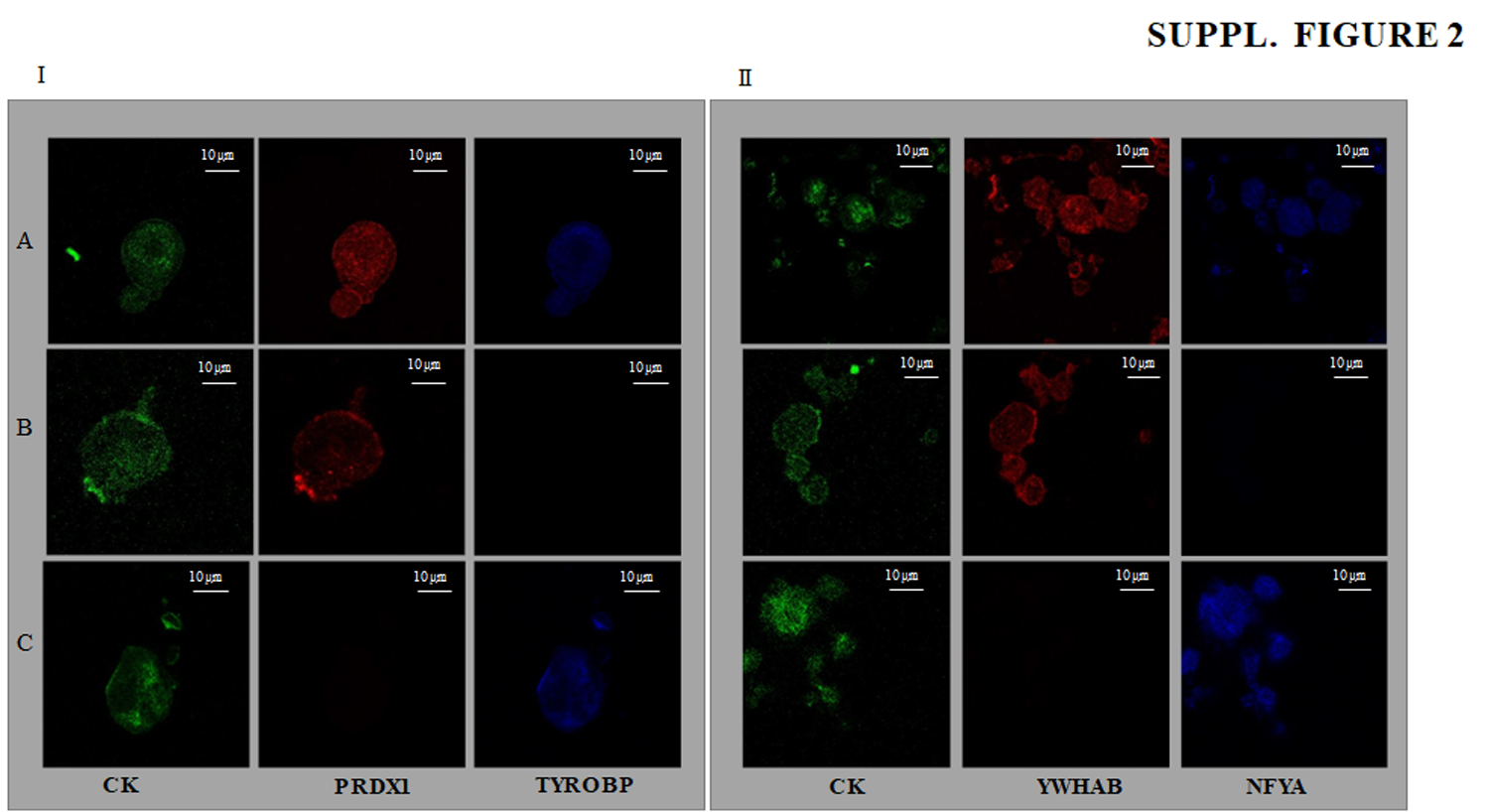

Supplement: Supplementary file 2 — Figure S2. (IA) Positive controls for CK/TYROBP/PRDX1 staining: MDA-MB 231 cells were stained with CK (green), TYROBP (blue), PRDX1 (red) antibodies, and the corresponding fluorochromes. (IB) Negative controls for CK/TYROBP/PRDX1 staining: Cells were stained with all the corresponding antibodies except the primary TYROBP antibody. (IC) Negative controls for CK/TYROBP/PRDX1 staining: Cells were stained with all the corresponding antibodies except the primary PRDX1 antibody. (IIA) Positive controls for CK/NFYA/YWHAB staining: MDA-MB 231 cells were stained with CK (green), NFYA (blue), YWHAB (red) antibodies, and the corresponding fluorochromes. (IIB) Negative controls for CK/NFYA/YWHAB staining: Cells were stained with all the corresponding antibodies except the primary NFYA antibody. (IIC) Negative controls for CK/NFYA/YWHAB staining: Cells were stained with all the corresponding antibodies except the primary YWHAB antibody. (TIF 4741 kb) [file 13058_2019_1166_MOESM2_ESM.tif]
